# Brain Entropy Study on Obsessive-Compulsive Disorder Using Resting-State fMRI

**DOI:** 10.3389/fpsyt.2021.764328

**Published:** 2021-11-12

**Authors:** Xi Jiang, Xue Li, Haoyang Xing, Xiaoqi Huang, Xin Xu, Jing Li

**Affiliations:** ^1^Magnetic Resonance Research Center, West China Hospital, Sichuan University, Chengdu, China; ^2^School of Physics, Sichuan University, Chengdu, China

**Keywords:** brain entropy, approximate entropy, sample entropy, fuzzy entropy (FuzzyEn), fMRI, OCD

## Abstract

**Object:** Brain entropy is a potential index in the diagnosis of mental diseases, but there are some differences in different brain entropy calculation, which may bring confusion and difficulties to the application of brain entropy. Based on the resting-state function magnetic resonance imaging (fMRI) we analyzed the differences of the three main brain entropy in the statistical significance, including approximate entropy (ApEn), sample entropy (SampEn) and fuzzy entropy (FuzzyEn), and studied the physiological reasons behind the difference through comparing their performance on obsessive-compulsive disorder (OCD) and the healthy control (HC).

**Method:** We set patients with OCD as the experimental group and healthy subjects as the control group. The brain entropy of the OCD group and the HC are calculated, respectively, by voxel and AAL region. And then we analyzed the statistical differences of the three brain entropies between the patients and the control group. To compare the sensitivity and robustness of these three kinds of entropy, we also studied their performance by using certain signal mixed with noise.

**Result:** Compare with the control group, almost the whole brain's ApEn and FuzzyEn of OCD are larger significantly. Besides, there are more brain regions with obvious differences when using ApEn comparing to using FuzzyEn. There was no statistical difference between the SampEn of OCD and HC.

**Conclusion:** Brain entropy is a numerical index related to brain function and can be used as a supplementary biological index to evaluate brain state, which may be used as a reference for the diagnosis of mental illness. According to an analysis of certain signal mixed with noise, we conclude that FuzzyEn is more accurate considering sensitivity, stability and robustness of entropy.

## Introduction

Entropy is a physical concept proposed by the German physicist Clausius in 1865, which is used to measure the complexity, randomness, or predictability of a dynamic process. In 1948, Shannon (1) introduced entropy into information theory and used entropy to quantify the complexity of information for the first time. And then the concept and application scope of entropy were gradually extended to cybernetics, probability theory, number theory, astrophysics, life science and other fields. Wang et al. (2) calculated the three-dimensional brain entropy map through the resting-state fMRI images of 1,049 subjects, and found that the distribution of brain entropy was consistent with the brain structure and functional partition. Saxe et al. (3) found that high intelligence would correspond to a high level of brain entropy in a sample of 892 healthy adults who participated in both resting-state fMRI and intelligence testing. Shi et al. (4) found that there was a significant positive relationship between creativity and the brain entropy values of DLPFC and DACC brain functional areas, which are responsible for cognitive flexibility and inhibitory control. Their study provides evidence of the associations of regional brain entropy with individual variations in divergent thinking and show that brain entropy is sensitive to detecting variations in important cognitive abilities in healthy subjects. Song et al. (5) found that brain entropy can be enhanced through caffeine intake, this study verifies the sensitivity of brain entropy to drug regulation, supports that brain entropy can sensitively reflect the neural effects of caffeine, and supports that brain entropy can be used as an indicator to detect changes in brain activity. Other researchers also reported changes in entropy in brain conditions such as normal aging (6–9), multiple sclerosis (10), schizophrenia (11), Alzheimer's disease (12), and Attention deficit hyperactivity disorder (13). These studies show that brain entropy can reflect information of the brain activity, and can be used as a tool for diagnosis and treatment of brain diseases, and can provide a potential way to explore complex brain function. There are three main types of brain entropy including ApEn, SampEn, and FuzzyEn, and they are certainly different among different brain entropy calculations. Up to now, the accuracy and sensitivity of the three different calculation methods in measuring the degree of brain dysfunction are not clear.

According to the Diagnostic and Statistical Manual of Mental Disorders V (DSM-V), OCD has become an independent disease with obsessive thinking and compulsive behavior as the main clinical manifestations. It is characterized by the coexistence of conscious compulsion and anti-compulsion, and some meaningless or even against one's own wishes. Impulse repeatedly invades the patient's daily life. Previous studies showed that there are Amplitude of Low Frequency Fluctuations (ALFF) abnormalities (14, 15) and Functional Connectivity (FC) abnormalities (16, 17) in the brains of OCD, and the neuron activity in the corresponding brain area [such as CSTC (18–20), DLPFC (21)] is stronger. These studies indicate that OCD may be a disease of brain abnormalities. However, for the study of abnormal brain areas and their functional status in OCD, the results of brain dysfunction areas in OCD detected by ALFF and FC are not consistent. Therefore, a more comprehensive, accurate and sensitive detection method is needed. The change of brain entropy can reflect the intensity of neuronal activity in the corresponding brain area, which can be used as a tool for the diagnosis and treatment of brain diseases. However, the relationship between abnormal neuron activity and brain entropy in OCD is still unclear.

In order to clarify the response of brain entropy to the brain function activities of OCD patients, three different brain entropies (ApEn, SampEn, and FuzzyEn) and their degree of response to OCD will be obtained in this experiment explore, and then elicit a new method of OCD diagnosis to provide experimental basis for further research on the pathogenesis of OCD.

### Definition of three Entropies

#### Approximate Entropy

ApEn is defined by Pincus (22) according to Kolmogorov entropy (23, 24), which is the conditional probability that the similarity vector will continue to maintain its similarity when it increases from *m* dimension to *m* + 1 dimension. Its physical meaning is the probability of the time series generating new patterns when the dimension changes. The greater the probability of the new pattern generated, the more complex the sequence is, the larger the corresponding ApEn is. It is a non-negative number which is used to quantify the regularity, unpredictability, and complexity of a time series. The calculation method is as follows:

Denote the rsfMRI data extract the time series of a voxel X= [*X*_1_, *X*_2_, …, *X*_*n*_]. Define the parameters *m* and *r*, where *m* is the pre-defined dimension, and *r* is a pre-specified distance threshold. Reconstruct the *m*-dimensional vector, *U*_*i*_ = [*X*_*i*_, *X*_*i*+1_, …, *X*_*i*+*m*−1_], where if = 1 *to n* − *m* + 1

The distance between the two vectors *d*(*U*_*i*_, *U*_*j*_) = *max*|*U*_*i*_(*a*)−*U*_*j*_(*a*)|, and we get


(1) $$\begin{array}{l} C_{i}^{m}\left(r\right)={\left(n-m+1\right)}^{-1}\left[the\;number\;of\text{ d}\left(U_{i},U_{j}\right)<r\right] \end{array}$$

(2) $$\begin{array}{l} \Phi^{m}\left(r\right)={\left(n-m+1\right)}^{-1}\sum_{i=1}^{N-m+1}C_{i}^{m}(r) \end{array}$$

Repeat the above steps to get Φ^*m* + 1^(*r*).


(3) $$\begin{array}{l} ApEn\left(m,r\right)=\Phi^{m}\left(r\right)-\;\Phi^{m+1}\left(r\right) \end{array}$$

#### Sample Entropy

SampEn (25) excludes self-matching compared to ApEn.


(4) $$\begin{array}{l} C_{i}^{m}\left(r\right)={\left(n-m-1\right)}^{-1}\left[the\;number\;of\text{d}\left(U_{i},U_{j}\right)<r\right] \end{array}$$

where *j* changes from 1 to n–m, and *j*≠*i*.


(5) $$\begin{array}{l} \Phi^{m}\left(r\right)={\left(n-m\right)}^{-1}\sum_{i=1}^{N-m+1}C_{i}^{m}(r) \end{array}$$

(6) $$\begin{array}{l} SampEn\left(m,r\right)=In\Phi^{m+1}\left(r\right)-\;In\Phi^{m}\left(r\right)=-\ln\left(\frac{\Phi^{m}\left(r\right)}{\phi^{m+1}\left(r\right)}\right) \end{array}$$

#### Fuzzy Entropy

FuzzyEn (26) introduces the fuzzy membership function μ(d) on the basis of the SampEn, and removes a baseline. Introduce the fuzzy membership function *u*(*d*):


(7) $$\begin{array}{l} u\left(d\right)=\{\begin{array}{cc} 1 & d=0\\ \exp\left[-ln\left(2\right){\left(\frac{d}{r}\right)}^{2}\right] & d>0 \end{array} \end{array}$$

Where d is the distance between the two vectors.


(8) $$\begin{array}{l} C_{i}^{m}\left(r\right)=\sum u\left(d\right)/\left(n-m+1\right) \end{array}$$

(9) $$\begin{array}{l} \Phi^{m}\left(r\right)={\left(n-m+1\right)}^{-1}\overset{N-m+1}{\underset{i=1}{\sum }}C_{i}^{m}\left(r\right) \end{array}$$

Repeat the above steps to get Φ^*m*+1^(*r*).


(10) $$\begin{array}{l} \text{FuzzyEn}\left(m,r\right)=\text{I}n\Phi^{m}\left(r\right)-\;In\Phi^{m+1}\left(r\right). \end{array}$$

### Comparison of Three Entropies

For ApEn:


(11) $$\begin{array}{l} \text{ApEn}\left(\text{m},\text{r}\right)=\Phi^{\text{m}}\left(\text{r}\right)-\Phi^{\text{m+1}}\left(\text{r}\right)\\ \;=\sum_{\text{i=1}}^{\text{N-m+1}}\ln[{\text{C}}_{\text{i}}^{\text{m}}\left(\text{r}\right)/{\text{C}}_{\text{i}}^{\text{m+1}}(\text{r})] \end{array}$$

Where pi$=C_{i}^{m}\left(r\right)/C_{i}^{m+1}\left(r\right)$ is the conditional probability that the time series will produce a new pattern when the dimension changes. In order to avoiding the appearance of ln(0), the ApEn has a self-comparison value in the process of comparing whether two vectors are similar. Obviously, such an algorithm is unscientific, so SampEn optimizes it and eliminates the bias caused by self-matching.

For SampEn:


(12) $$\begin{array}{l} SampEn\left(m,r\right)=\text{In}\Phi^{\text{m}}\left(\text{r}\right)-\text{In}\Phi^{\text{m+1}}\left(\text{r}\right)\\ \;=\text{In}[\sum {\text{C}}_{\text{i}}^{\text{m}}\left(\text{r}\right)/\sum {\text{C}}_{\text{i}}^{\text{m+1}}\left(\text{r}\right)] \end{array}$$

The calculation of SampEn is to first sum and then take the logarithm to avoid the appearance of ln(0), which does not include the comparison of its own data segments. The other steps are similar to ApEn, so SampEn theoretically has higher accuracy than ApEn. Although SampEn is an improved algorithm of ApEn, ApEn is closely related to the definition of the traditional definition of entropy whose existence helps us better understand the nature of entropy.

For ApEn and SampEn, judging whether two vectors are similar is depending on the parameter *r*. As long as the distance is within *r*, the two vectors are considered similar, which means that they will be considered dissimilar even if the distance is only slightly larger than *r*. That will let them have significant changes due to changes in the parameter *r*. For FuzzyEn, the concept of fuzzy is introduced into the calculation of entropy. The value taken in the interval [0, 1] is used to replace *t* 0 and 1. The soft continuous boundary not only guarantees the definition of the fuzzy function at small parameters, but also makes the fuzzy function change continuously. In addition, compared to ApEn and FuzzyEn, when constructing the m-dimensional similarity vector, a baseline is removed, which makes the definition of FuzzyEn more accurate than the other two (26).

## Materials and Methods

### Subjects

We recruited 74 drug-naive patients diagnosed with OCD using the Structured Clinical Interview for DSM-IV Axis I Disorders (SCID) by two experienced psychiatrists from the Mental Health Center, West China Hospital, Sichuan University, and 93 healthy control subjects (HCs) matched for sex and age via poster advertisements. Only right-handed Chinese individuals between 18 and 60 years of age were included, and the exclusion criteria for both groups included (1) any history of major physical illness, cardiovascular disease, or psychiatric or neurological disorder; (2) substance abuse or dependence; (3) inability to undergo an MRI scan; and (4) pregnancy. Additionally, OCD patients with a psychiatric comorbidity assessed using the SCID were excluded. The Yale-Brown-Obsessive-Compulsive Scale (YBOCS) was used to evaluate OCD symptoms severity, and anxiety and depressive symptoms were assessed using the 14-item Hamilton Anxiety Scale (HAMA) and 17-item Hamilton Depression Scale (HAMD), respectively. This study was approved by the Ethics Committee of the West China Hospital, Sichuan University. All subjects provided an informed consent form.

### rsfMRI Data Acquisition and Preprocessing

All the images were obtained using a 3T GE MRI scanner with an eight-channel phase-array head coil. The following scanning parameters were used: number of slices = 30, time repetition (TR) = 2,000 ms, time echo = 30 mms, flip angle = 90, slice thickness = 5 mm with no slice gap, field of view = 240 × 240 *mm*^2^, and 200 volumes in each run. We also acquired a high-resolution *T*_1_-weighted 3D sequence (TR = 8.5ms, echo time = 3.4 ms, flip angle = 12), slice thickness = 1.0mm, field of view = 240 × 240 *mm*^2^). Imaging preprocessing was carried out using the Data Processing and Analysis of Brain Imaging (DPABI) toolbox (27) (http://rfmri.org/dpabi). Preprocessing steps included (1) discarding the first 10 images for magnetization equilibrium and (2) slice timing correction and head-motion correction for the remaining 190 images. The mean framewise displacement (FD) was calculated to evaluate the head movement of each participant. To minimize the effect of head motion, we selected a stringent criterion: excluded the participants whose maximal head movement translation exceeded 3 mm, whose mean FD was more than 0.2 mm or whose rotation was more than 2. One patient and three HCs were excluded due to excessive head motion. Next, (3) spatial normalization to standard Montreal Neurological Institute space and resampling to 3 × 3 × 3 *mm*^3^ resolution *via* *T*_1_-weighted anatomical images were performed. (4) The cerebrospinal fluid signal, white matter and Friston-24 motion parameters were considered nuisance covariates, and global signal regression was not used (28). Finally, (5) the data were spatially smoothed with an 8 mm full-width half-maximum (FWHM) isotropic Gaussian kernel.

### Brain Entropy Calculation

After preprocessing, we calculated the brain entropy. Refer to 1.1 for the calculation method, and based on the literature (26), *m* usually is taken as 2 or 3, and *r* is usually taken from 0.2 to 1.2, which depends on the actual application scenario. In this experiment we take *m* = 3 and *r* = 0.2.

### Statistical Analysis

Two-sample *t*-test was used to compare the differences in brain entropy between the two groups. The covariables contained age, gender, and head motion. And we used false discover rate (FDR) correction for the multiple comparisons and the significance level was set *p* < 0.05.

## Result

### The Distribution of Brain Entropy

**Figure 1:**
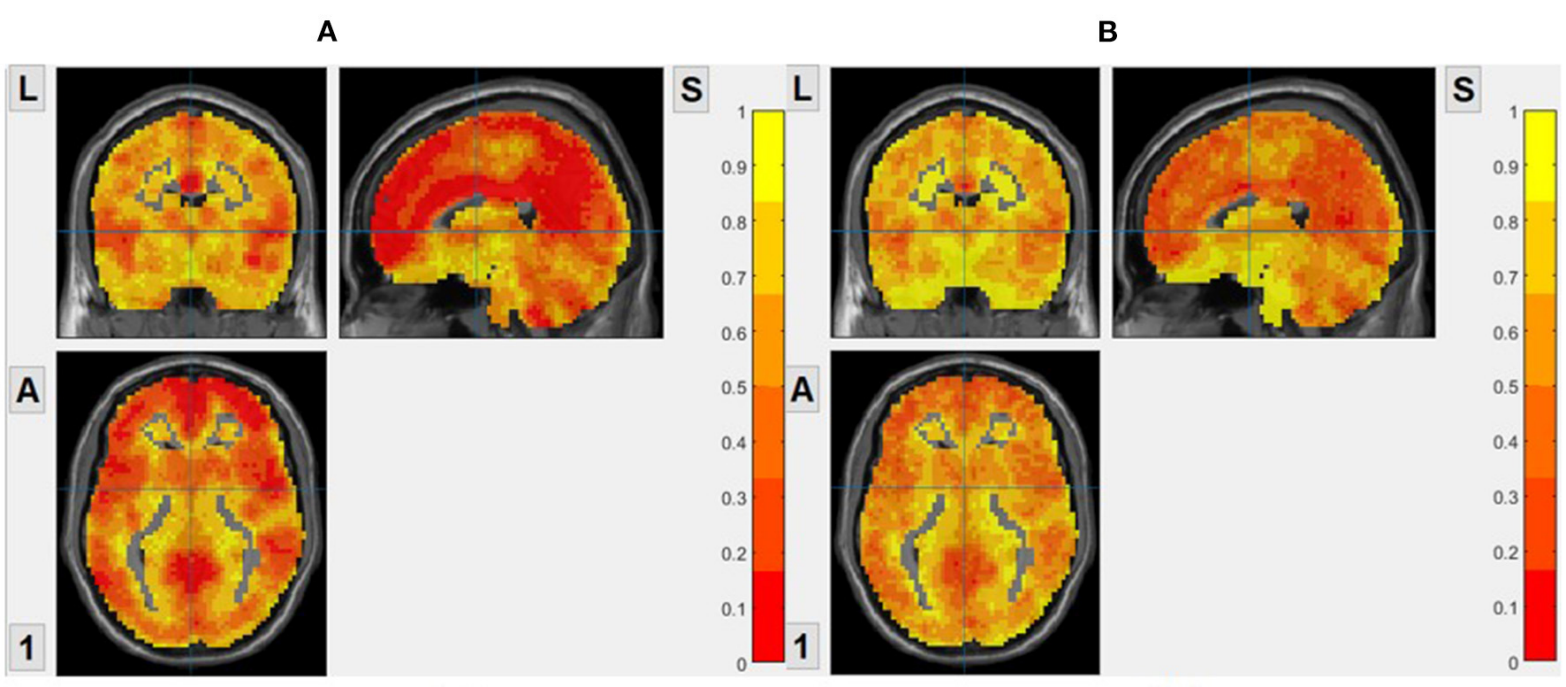
The brain entropy distribution of a normal subject. **(A)** ApEn, based on voxel, *m* = 2, *r* = 0.2. **(B)** FuzzyEn, based on voxel, *m* = 2, *r* = 0.2.

As shown in Figure 1, We calculated the brain entropy of the entire brain area of normal people and found that the distribution of ApEn and FuzzyEn is generally consistent with the brain structure and functional partition, but SampEn isn't. So, we do not show the brain entropy distribution of SampEn.

### Gender Differences of Brain Entropy

We divided the subjects in the group into two groups according to gender, and compare whether there are obvious gender differences in brain entropy. None of the three entropies show significant differences between genders.

### Brain Entropy Based on Voxel

**Figure 2:**
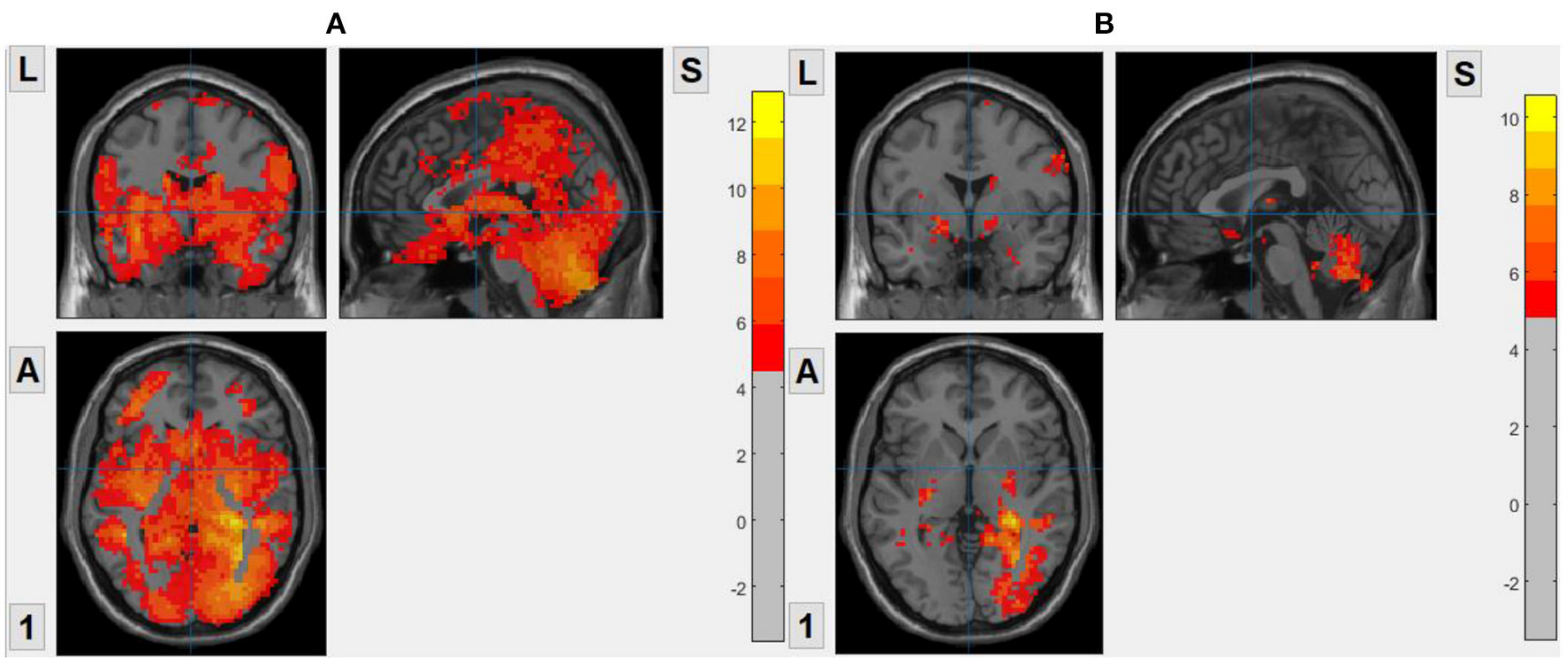
Brain regions with statistical differences between OCD and HC. Warm color means OCD has a higher value. **(A)** ApEn, based on voxel, *m* = 2, *r* = 0.2. **(B)** FuzzyEn, based on voxel, *m* = 2, *r* = 0.2.

We set patients with OCD as the experimental group and healthy subjects as the control group. The brain entropy of the OCD group and the HC are calculated by voxel. And we use two-sample t-test to compare the differences in brain entropy between the two groups. The covariables contained age, gender, and head motion. And we used false discover rate (FDR) correction for multiple comparisons and the significance level was set *p* < 0.05. The result is shown in Figure 2, For ApEn, the result shows that the ApEn of almost the whole brain of OCD is greater than that of HC. For FuzzyEn, the result also shows that the FuzzyEn of OCD is greater than that of HC, but there are fewer brain regions with difference. For SampEn, there is no statistical difference between OCD and HC, so we do not show that.

### Brain Entropy Based on AAL

**Figure 3:**
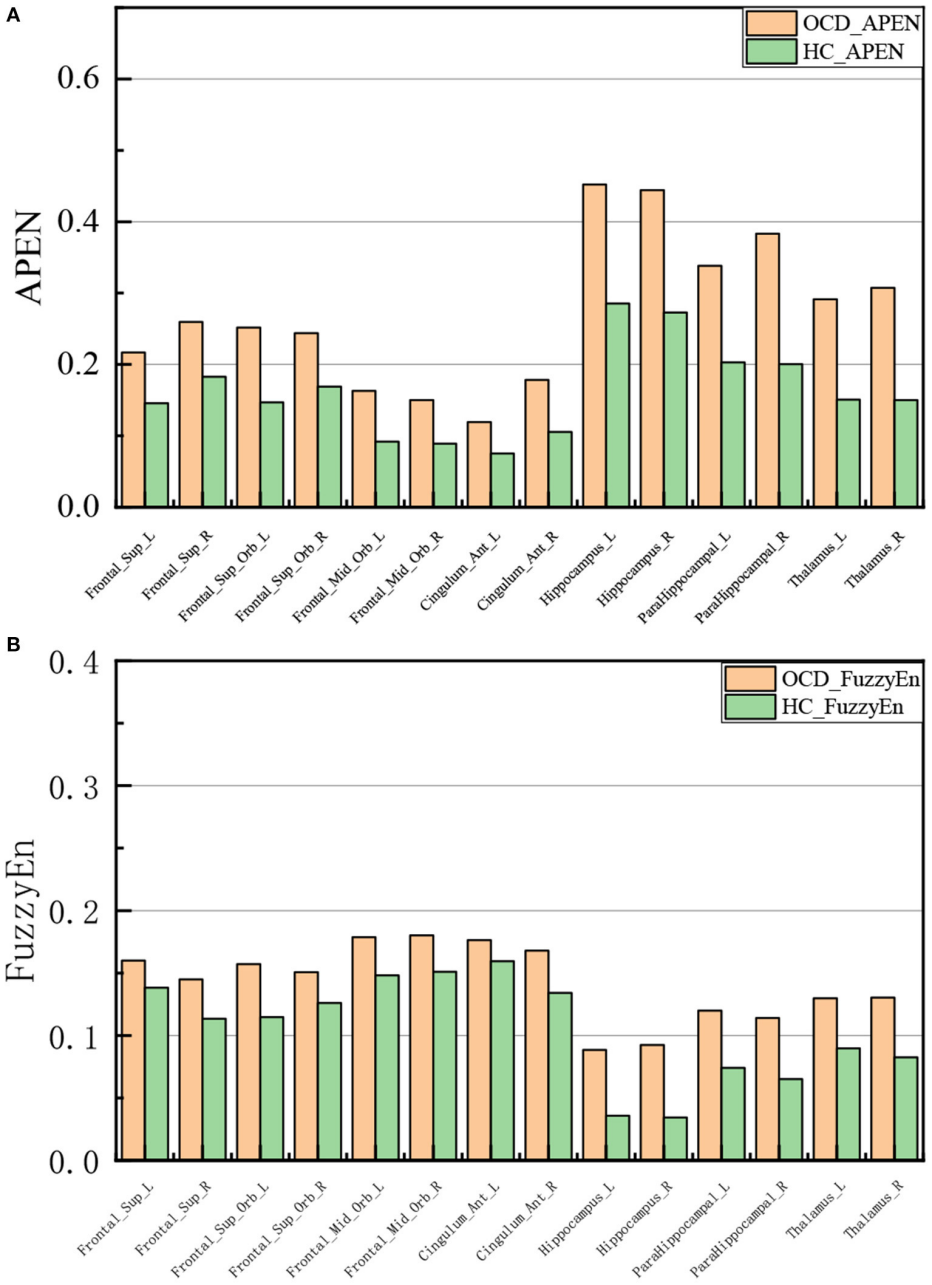
Brain regions with statistical differences between OCD and HC. **(A)** ApEn **(B)** FuzzyEn.

We took the average of the time series of all voxels in the same brain area as the time series of the brain area, and then calculated the brain entropy values based on AAL. Figure 3 shows the brain regions with a significant difference in brain entropy between OCD and HC including frontal region, Hippocampus, ParaHippocampal, and Thalamus.

## Discussion

From this experiment, both ApEn and FuzzyEn can reflect the abnormality of OCD brain entropy. And it is obvious that compared with FuzzyEn there are more different brain regions using ApEn. It seems that ApEn is more sensitive and can better reflect the difference between OCD and HC, which is not in accordance with the results of our previous theoretical analysis (the definition of FuzzyEn is more accurate than the other two).

In order to further evaluate the reliability of the different brain entropy analysis, we calculate the entropy of a simulated signal consisting of a certain signal and noise to investigate their robustness. Details and results are as follows.

**Figure 4:**
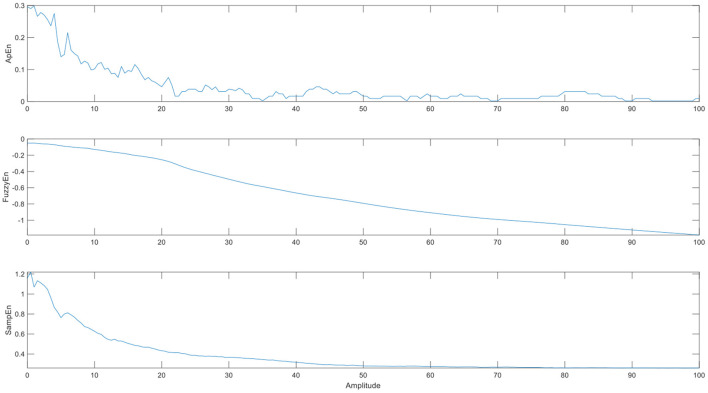
The brain entropy adding different amplitudes of sine waves to a voxel time series of cerebrospinal fluid. (The abscissa is the amplitude of the sine wave, and its value is a multiple of the maximum noise).

To test the sensitivity of the three entropies, we extract a voxel time series of cerebrospinal fluid as noise, add sine waves of different amplitudes as certain signals, and then measure the brain entropy of the mixed signal. From Figure 4, we can see that the entropy decreases with the amplitude of the sine wave increases. When the signal amplitude is much greater than the noise, the entropy is almost a fixed value. The change of SampEn and ApEn is faster than FuzzyEn, which maybe affect the sensitivity of entropy index, but the ApEn is unstable and fluctuates irregularly.

**Figure 5:**
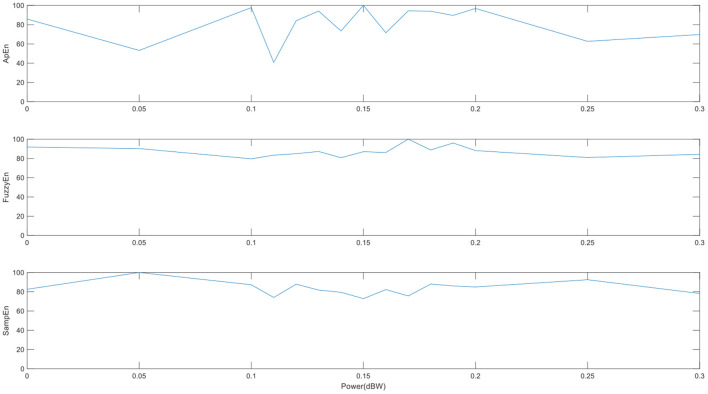
The performances of the three entropies adding different noise level. (The abscissa is the intensity of the specified output noise in the unit).

To test the robustness to noise of the three entropies, we measure the brain entropy of the sine wave signal, which add Gaussian white noise with different intensity of noise. From Figure 5, it can be found that ApEn fluctuates greatly and is unstable with the change of noise intensity, which may be indicate that ApEn shows quite sensitive to the changes of noise, while FuzzyEn is the most stable to the change in noise. FuzzyEn shows the best robustness to noise, which is similar to the results of previous studies (26).

From the above two simulations, we find that although ApEn has higher sensitivity, its robustness to noise is not as good as FuzzyEn. This may be the reason why we get more brain regions with entropy difference between OCD and HC when we use ApEn. In addition, although SampEn shows good sensitivity and robustness to noise in the simulation, in the experiment, it does not show the difference between OCD and HC. This may be because the above two simple simulations cannot fully evaluate these three entropies, and it may also be related to the selection of parameters *r*. When we take similarity *r* = 0.6, there is a significant difference between OCD and HC. So, we integrated theory, experiment, and simulation to conclude that FuzzyEn is the most accurate.

There are studies finding that the onset of OCD is probably due to the abnormality of the Cortico-striate-thalami-cortical (CSTC) (18), especially the orbit frontal cortex (OFC), anterior cingulate cortex (ACC), striatum and thalamus (20). In addition, studies have pointed out that the brain default network (29), the prominent network and the limbic system of OCD patients are also abnormal to a certain extent. And, in this article, compare with the control group, almost the whole brain's entropies of OCD are larger significantly. Among them the entropy of the default network (default mode network, DMN), left and right OFC, thalamus, and ACC increase the most. OFC is a classic brain area in the CSTC loop. Previous studies have shown that it has abnormalities in structure, function, and metabolism. ACC is involved in the regulation of selective attention; OFC is involved in the regulation of impulsive behavior. Entropy represents the irregularity and information processing ability of a system, and the increase of entropy indicates the increase of the randomness and complexity of a system, which means that OCD patients perform an increased neuronal activity in the above brain areas in the resting state. And this is also consistent with the clinical manifestations of OCD obsessive thinking and compulsive behavior. The abnormality of CTSC and DMN function was verified from the perspective of entropy, which is similar to the results of previous studies.

In this study, the FuzzyEn of the left dorsolateral prefrontal cortex (DLPFC) of patients with OCD in the resting state is significantly higher. DLPFC participates in anxiety and other negative emotion regulation (30, 31) and goal-oriented planning, cognitive re-evaluation, and other cognitive executive functions (32), which is consistent with our brain entropy research results. The hippocampus is an important emotion regulation center, and has always been an important nucleus of depression research. And the paraHippocampal gyrus is an important structure for the hippocampus to function. Damage to its structure can cause abnormalities in emotion and cognitive behavior. This study found that the fuzzy entropy of the above two brain regions of OCD patients was significantly greater than that of HC.

There are still a few limitations in this study. The sample size is generally small, and the conclusions are not very representative. Secondly the fMRI data has the characteristics of low signal-to-noise ratio. And we did not consider the influence of the characteristic symptoms of OCD patients on neuronal activity during the statistical analysis. The above two simple simulations cannot fully evaluate these three entropies. Our future research plans to further increase the sample size, refine the characteristic symptoms, eliminate the noise, and simulate the real BOLD signal as much as possible to study the brain conditions of OCD.

## Conclusion

Brain entropy can quantitatively describe non-linear time series and provide new ideas for describing brain characteristics. From a theoretical point of view, the core of these three types of entropy is the conditional probability that the similarity vector continues to maintain its similarity when it increases from m-dimension to m + 1-dimension. The optimization of SampEn relative to ApEn is to exclude the influence of self-matching. The optimization of FuzzyEn lies in the introduction of a fuzzy boundary, which makes it less sensitive to the distance threshold *r*, and a slight change in *r* will not change the result greatly.

From the experimental results, although ApEn has higher sensitivity, its robustness to noise is not as good as FuzzyEn. And the SampEn does not show the difference between OCD and HC. we conclude that FuzzyEn is more accurate considering sensitivity, stability and robustness of entropy.

In conclusion, the brain is a complex system, and brain entropy can measure the complexity of the human brain. It provides us with different aspect for researching brain activity, and helps us understand our brains in a different direction. In addition, among the three kinds of entropy mentioned in the article, both theoretically and experimentally, it seems that using FuzzyEn is more accurate.

## Data Availability Statement

The original contributions presented in the study are included in the article/supplementary material, further inquiries can be directed to the corresponding author/s.

## Ethics Statement

The studies involving human participants were reviewed and approved by the Ethics Committee of the West China Hospital, Sichuan University. The patients/participants provided their written informed consent to participate in this study.

## Author Contributions

XJ: conceptualization, formal analysis, methodology, software, visualization, writing—original draft, writing—review and editing. XL: conceptualization, data curation, formal analysis, methodology, validation, writing—review and editing. HX and XH: writing—review and editing, funding acquisition, project administration, and supervision. XX and JL: writing—review and editing. All authors contributed to the article and approved the submitted version.

## Funding

This study was supported by the National Key Technologies R&D Program of China (Grant No. 82027808), the Key Research Project of Sichuan Science and Technology Department (Grant No. 2020YFS0048), and the Science Specialty Program of Sichuan University (Grant No. 2020SCUNL210).

## Conflict of Interest

The authors declare that the research was conducted in the absence of any commercial or financial relationships that could be construed as a potential conflict of interest.

## Publisher's Note

All claims expressed in this article are solely those of the authors and do not necessarily represent those of their affiliated organizations, or those of the publisher, the editors and the reviewers. Any product that may be evaluated in this article, or claim that may be made by its manufacturer, is not guaranteed or endorsed by the publisher.
